# Polysaccharide from Edible Alga *Enteromorpha clathrata* Improves Ulcerative Colitis in Association with Increased Abundance of *Parabacteroides* spp. in the Gut Microbiota of Dextran Sulfate Sodium-Fed Mice

**DOI:** 10.3390/md20120764

**Published:** 2022-12-04

**Authors:** Mingfeng Ma, Tianyu Fu, Yamin Wang, Aijun Zhang, Puyue Gao, Qingsen Shang, Guangli Yu

**Affiliations:** 1 Key Laboratory of Marine Drugs of Ministry of Education, Shandong Key Laboratory of Glycoscience and Glycotechnology, School of Medicine and Pharmacy, Ocean University of China, Qingdao 266003, China; 2 Laboratory for Marine Drugs and Bioproducts, Qingdao National Laboratory for Marine Science and Technology, Qingdao 266003, China; 3 Qilu Hospital of Shandong University (Qingdao), Qingdao 266035, China; 4 Qingdao Marine Biomedical Research Institute, Qingdao 266071, China

**Keywords:** *Enteromorpha clathrata*, polysaccharide, ulcerative colitis, *Parabacteroides distasonis*, dextran sulfate sodium, inflammatory bowel disease, short-chain fatty acids

## Abstract

Polysaccharide from the edible alga *Enteromorpha clathrata* has been demonstrated to exert beneficial effects on human health. However, what effect it has on inflammatory bowel diseases has not been investigated. Here, using a mouse model of dextran sulfate sodium (DSS)-induced ulcerative colitis, we illustrate that *Enteromorpha clathrata* polysaccharide (ECP) could alleviate body weight loss, reduce incidences of colonic bleeding, improve stool consistency and ameliorate mucosal damage in diseased mice. 16S rRNA high-throughput sequencing and bioinformatic analysis indicated that ECP significantly changed the structure of the gut microbiota and increased the abundance of *Parabacteroides* spp. in DSS-fed mice. In vitro fermentation studies further confirmed that ECP could promote the growth of *Parabacteroides distasonis* F1-28, a next-generation probiotic bacterium isolated from the human gut, and increase its production of short-chain fatty acids. Additionally, *Parabacteroides distasonis* F1-28 was also found to have anti-ulcerative colitis effects in DSS-fed mice. Altogether, our study demonstrates for the first time a beneficial effect of ECP on ulcerative colitis and provides a possible basis for understanding its therapeutic mechanisms from the perspective of symbiotic gut bacteria *Parabacteroides distasonis*.

## 1. Introduction

*Enteromorpha clathrata* is an edible alga that has been widely consumed in Asian countries [1,2,3,4]. Previous studies have indicated that *Enteromorpha clathrata* polysaccharide (ECP) could be used as a novel drug candidate to treat obesity, constipation and hyperlipidemia [3,5,6]. However, what effect it has on inflammatory bowel diseases has not been investigated. Ulcerative colitis and Crohn’s disease are two types of typical inflammatory bowel diseases, and preceding results have illustrated a causal role of gut dysbiosis in the development of ulcerative colitis [7,8,9,10]. ECP is not absorbed after oral intake and therefore holds great potential for the treatment of dysbiosis-associated intestinal diseases by targeting gut microbiota [1,2,4].

Our previous results suggested that dietary intake of ECP could modulate the composition of the gut microbiota and increase the abundances of beneficial microbes in the gut, including *Akkermansia muciniphila*, *Eubacterium xylanophilum*, *Bifidobacterium* spp. and *Lactobacillus* spp. [1,5]. Similarly, Ren et al. found that ECP could ameliorate loperamide-induced gastrointestinal disorders by increasing the populations of probiotic bacteria such as *Prevotella* spp. and *Bacteroides* spp. in the colon [6]. Taken together, the prebiotic effect of ECP prompted us to investigate whether it also has any therapeutic effects on inflammatory bowel diseases. 

In the present study, using a mouse model of dextran sulfate sodium (DSS)-induced ulcerative colitis, we illustrate for the first time a beneficial effect of ECP on ulcerative colitis and provide a possible basis of understanding its therapeutic mechanisms from the perspective of symbiotic gut bacteria *Parabacteroides distasonis*. Our study paves the way for the development of ECP as a new drug candidate for the treatment of inflammatory bowel diseases.

## 2. Results

### 2.1. Dietary ECP Improved Ulcerative Colitis and Ameliorated Mucosal Damage in DSS-Fed Mice

**Figure 1:**
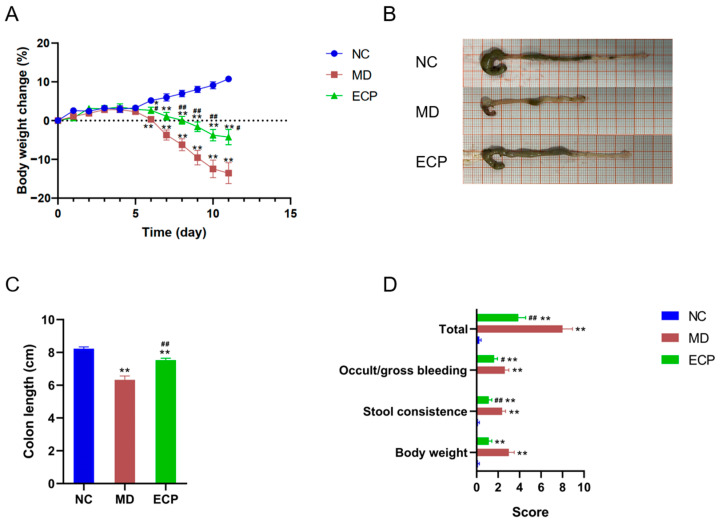
Dietary ECP improved ulcerative colitis in DSS-fed mice. Changes of the body weight during the experiment (**A**). Representative morphologies of the colon (**B**). Colon length (**C**). Symptom score analysis of ulcerative colitis (**D**). * *p* < 0.05 versus NC group; ** *p* < 0.01 versus NC group; ^#^ *p* < 0.05 versus MD group; ^##^ *p* < 0.01 versus MD group.

We explored the therapeutic effect of ECP on ulcerative colitis using a well-established mouse model [11,12]. DSS-induced ulcerative colitis in mice is associated with decreased body weight and shortening of the colon length. Interestingly, dietary intake of ECP (100 mg/kg) considerably retarded the body weight loss in DSS-treated mice (Figure 1A). Additionally, the shortening of colon length in diseased mice was also profoundly suppressed by the treatment of ECP (Figure 1B,C). Furthermore, oral administration of ECP also significantly reduced the incidences of colonic bleeding and improved stool consistency in diseased mice (Figure 1D).

**Figure 2:**
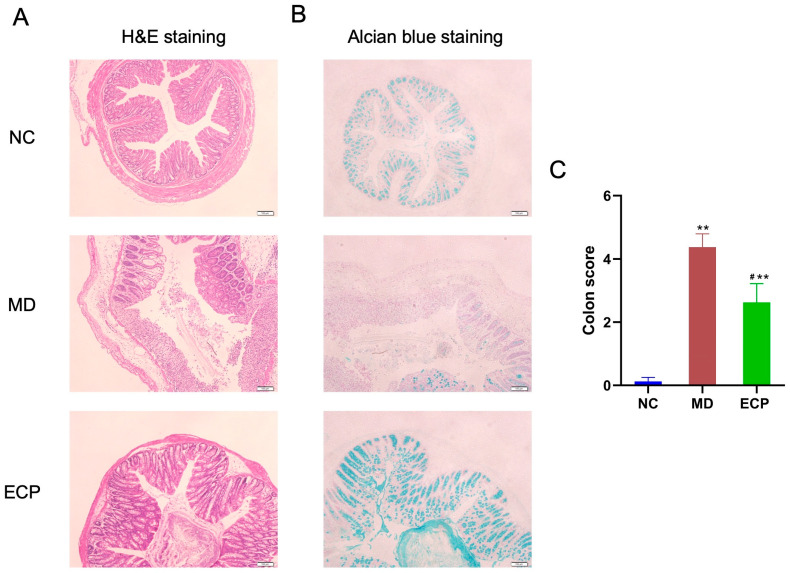
Dietary ECP ameliorated DSS-induced mucosal damage in the colon. H&E staining (**A**) and Alcian blue staining (**B**) of the colon tissues. Alcian blue staining was applied to show the changes of the intestinal acidic mucin *O*-glycans. Histopathological colon score analysis (**C**). ** *p* < 0.01 versus NC group; ^#^ *p* < 0.05 versus MD group.

Previous studies have indicated that DSS-induced ulcerative colitis is correlated with mucosal damage in the colon [11,12,13]. In line with previous results, DSS disrupted the intestinal epithelial layer and inhibited the biosynthesis of colonic mucin *O*-glycans (Figure 2A,B). However, intriguingly, dietary intake of ECP reversed the toxic effect of DSS and attenuated the mucosal damage in the colon of diseased mice, as indicated by histochemical staining (Figure 2A,B) and histopathological colitis score analysis (Figure 2C).

### 2.2. Dietary ECP Changed the Overall Structure of the Gut Microbiota in DSS-Fed mice

**Figure 3:**
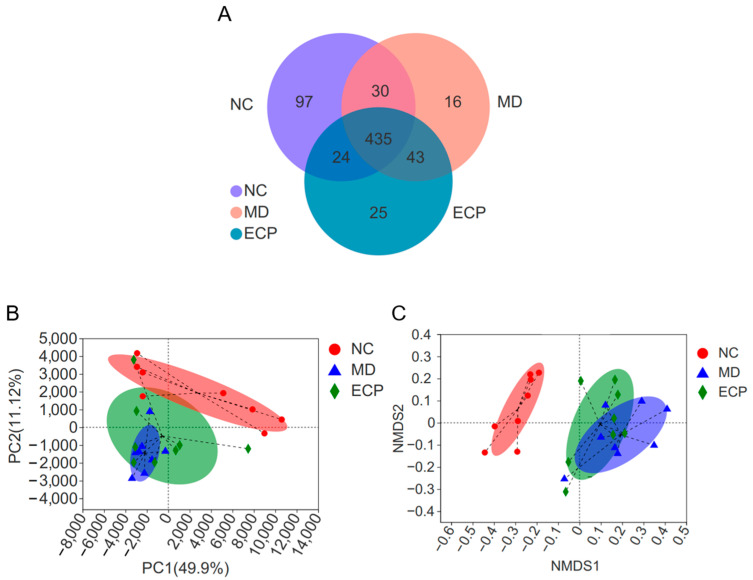
Dietary ECP changed the overall structure of the gut microbiota in diseased mice. Venn diagram analysis of the OTUs (**A**). PCA score plot analysis (**B**). NMDS score plot analysis (**C**).

Previous studies have indicated that ECP is not absorbed after oral intake [1,2,4]. Therefore, when reaching the colon, it would be fermented by the intestinal microbes [1,4,5]. In this regard, we next sought to explore the beneficial effects of ECP on ulcerative colitis from the perspective of gut microbiota. Expectedly, dietary ECP significantly changed the overall structure of the gut microbiota in DSS-fed mice. Venn diagram analysis suggested that 49 and 46 operational taxonomic units (OTUs) were separately identified in ECP-treated mice and diseased mice (Figure 3A). Additionally, as indicated by principal components analysis (PCA) and non-metric multidimensional scaling (NMDS) score plot analyses, dietary ECP induced a remarkable shift of gut microbiota structure towards that of healthy mice (Figure 3B,C).

### 2.3. Dietary ECP Modulated the Composition of the Gut Microbiota and Increased the Abundance of Parabacteroides spp. in DSS-Fed Mice

**Figure 4:**
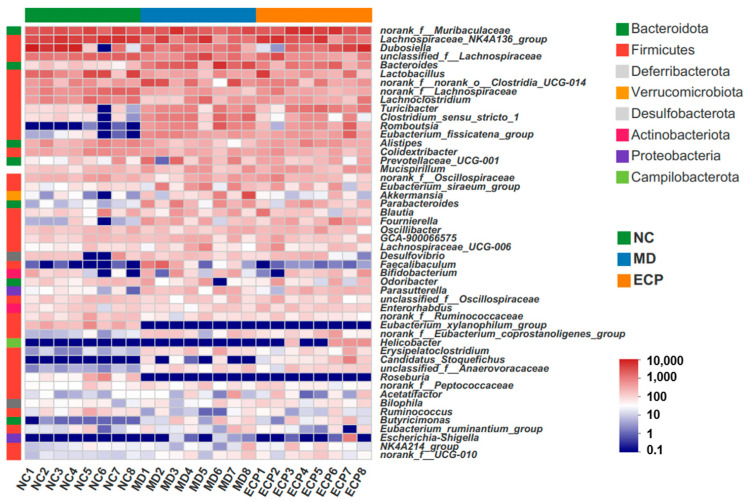
Dietary ECP modulated the composition of the gut microbiota at different taxonomic levels. Heatmap analysis of the gut microbiota at the phylum and genus levels.

The mice gut microbes were dominated by Bacteroidota, Actinobacteriota and Firmicutes at the phylum level and *Dubosiella* spp., *Bacteroides* spp., *Lactobacillus* spp., *Alistipes* spp., *Parabacteroides* spp. and *Blautia* spp. at the genus level (Figure 4). Heatmap analysis of the gut microbiota showed that intake of ECP could potentially affect the composition of the gut microbiota (Figure 4). 

**Figure 5:**
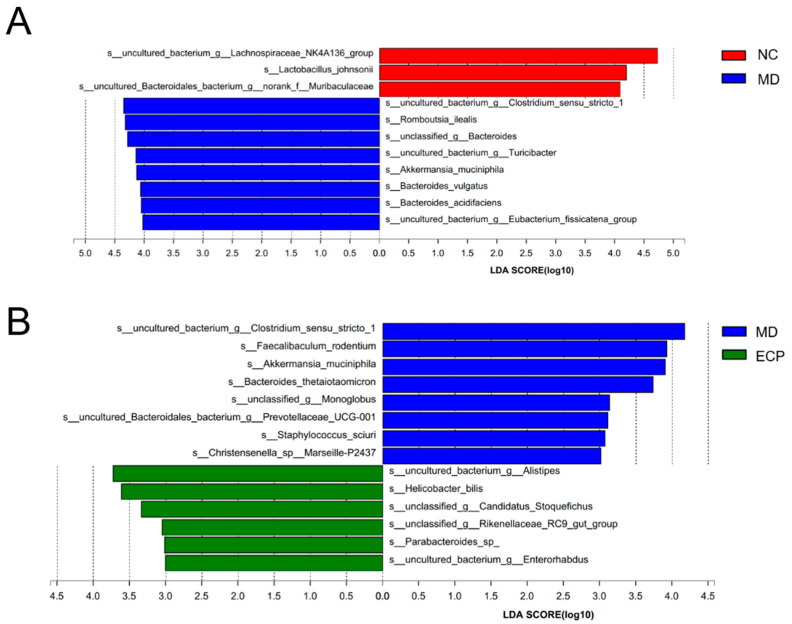
LEfSe LDA score analysis of the gut microbiota in NC versus MD group (**A**) and MD versus ECP group (**B**). Only bacterial taxa with an LDA score of above 4.0 and 3.0 are listed.

To identify the key bacteria that were changed by ECP treatment, we performed LEfSe analysis. NC mice were characterized by higher abundances of *Lachnospiraceae NK4A136* spp., *Lactobacillus johnsonii* and *Muribaculaceae* compared to DSS-fed mice (Figure 5A). In line with the preceding results [13], DSS-induced ulcerative colitis was associated with increased amounts of mucolytic bacteria, including *Akkermansia muciniphila*, *Bacteroides vulgatus*, *Bacteroides acidifaciens* and *Romboutsia ilealis* (Figure 5A). Interestingly, ECP treatment remarkably decreased the amount of mucolytic bacteria, including *Akkermansia muciniphila* and *Bacteroides thetaiotaomicron* and promoted the growth of potential beneficial bacteria such as *Parabacteroides* spp. and *Alistipes* spp. (Figure 5B). 

We next tried to isolate the probable colitis-protective bacteria in the genus of *Parabacteroides* spp. and *Alistipes* spp. from the colon contents of ECP-treated mice. However, we were not able to obtain the targeted bacteria. For this reason, we then turned to *Parabacteroides distasonis* F1-28, a bacterium that has been previoulsy isolated from the feces of a healthy individual in our lab. *P. distasonis* was chosen because previous studies have indicated that it could alleviates 2,4,6-trinitrobenzenesulfonic acid (TNBS)-induced colitis [14,15]. *P. distasonis* has been proposed as a next-generation probitic bacterium [14], and we hypothesized that ECP might be fermentated by *P. distasonis* F1-28, and *P. distasonis* F1-28 might also be protective against DSS-induced ulcerative colitis.

### 2.4. ECP Promoted the Growth of P. distasonis F1-28 In Vitro and Increased the Production of Short-Chain Fatty Acids

**Figure 6:**
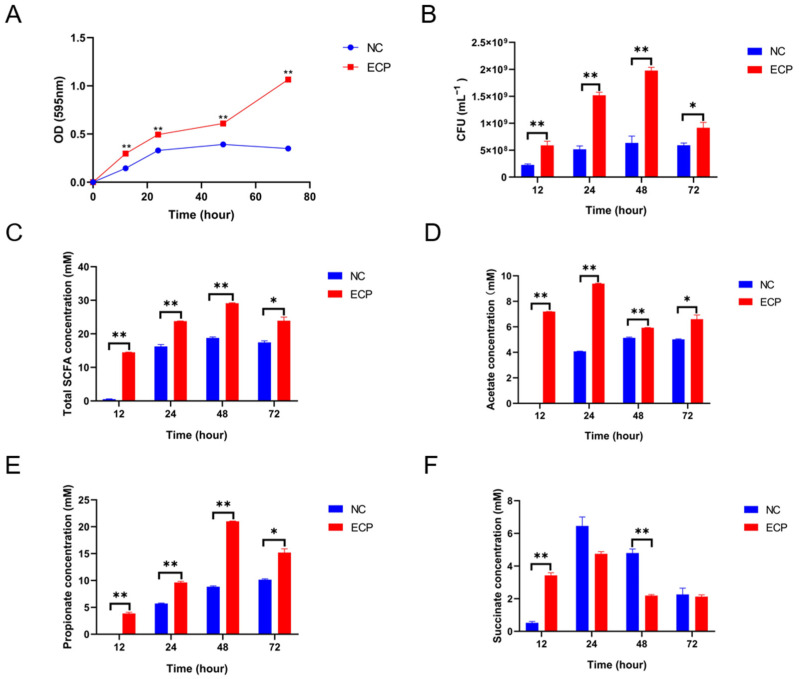
ECP promoted the growth of *P. distasonis* F1-28 and increased the production of SCFAs. Growth curves (**A**) and colony forming units (CFUs) (**B**). Concentrations of total SCFAs (**C**), acetate (**D**), propionate (**E**) and succinate (**F**). * *p* < 0.05 versus NC group; ** *p* < 0.01 versus NC group.

To test the aforementioned hypothesis, ECP was added to the culture medium of *P. distasonis* F1-28. Interestingly, ECP significantly promoted the growth of *P. distasonis* F1-28 in vitro (Figure 6A,B). Short-chain fatty acids (SCFAs), including acetate, propionate and succinate, are major fermentation products of *P. distasonis* [14]. In the present study, ECP considerably increased the productions of total SCFAs, acetate, propionate and succinate at different fermentation periods (Figure 6C–F). Altogether, our study indicates that ECP could be utilized and fermented by *P. distasonis* in the gut, and this could help to explain why the abundance of *Parabacteroides* spp. was increased in ECP-treated mice.

### 2.5. P. distasonis F1-28 Alleviated Ulcerative Colitis and Attenuated Mucosal Damage in DSS-Fed Mice

**Figure 7:**
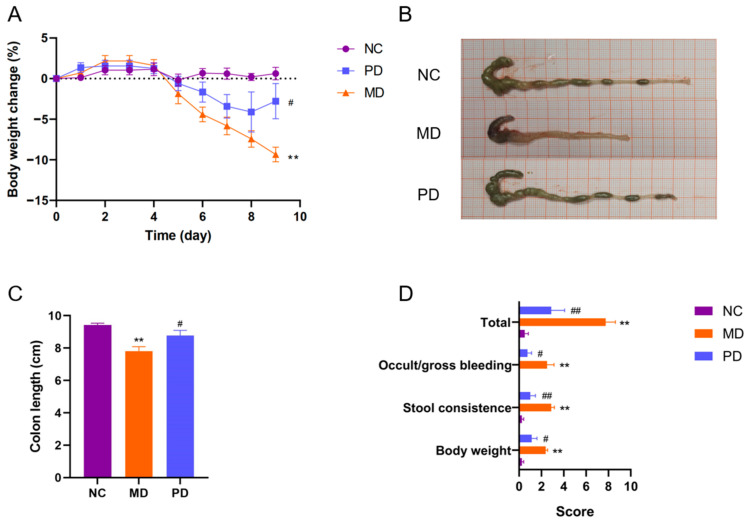
Oral administration of live *P. distasonis* F1-28 alleviated ulcerative colitis in DSS-fed mice. Changes of the body weight during the experiment (**A**). Representative morphologies of the colon (**B**). Colon length (**C**). Symptom score analysis of ulcerative colitis (**D**). ** *p* < 0.01 versus NC group; ^#^ *p* < 0.05 versus MD group; ^##^ *p* < 0.01 versus MD group.

In light of the fact that ECP promotes the growth of *P. distasonis* F1-28, we next sought to investigate whether *P. distasonis* F1-28 has any therapeutic effects on DSS-induced colitis. Interestingly, we found that oral administration of live *P. distasonis* F1-28 could alleviate ulcerative colitis in DSS-fed mice. Specifically, intake of *P. distasonis* F1-28 retarded the body weight loss, reduced the contraction of the colon length, decreased the incidences of bleeding and improved stool consistency in diseased mice (Figure 7A–D).

**Figure 8:**
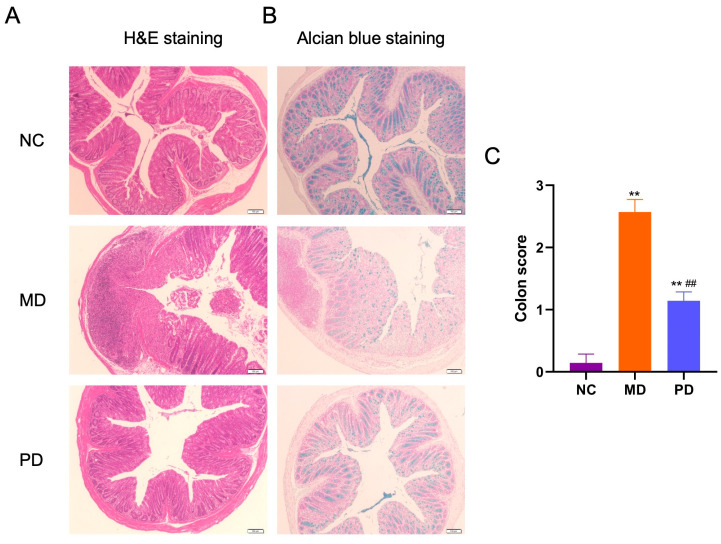
Oral administration of live *P. distasonis* F1-28 attenuated DSS-induced mucosal damage in the colon. H&E staining (**A**) and Alcian blue staining (**B**) of the colon tissues. Histopathological colon score analysis (**C**). ** *p* < 0.01 versus NC group; ^##^ *p* < 0.01 versus MD group.

Histochemical staining and histopathological colitis score analysis suggested that *P. distasonis* F1-28 could also attenuate DSS-induced mucosal damage in the colon (Figure 8A–C). Altogether, our study corroborates previous results showing an anti-inflammatory effect of *P. distasonis* on TNBS-induced colitis [15]. ECP could promote the growth of *P. distasonis* F1-28, and this could help to understand the therapeutic effect of ECP on chemical-induced colitis in mice.

## 3. Discussion

*Enteromorpha clathrata* is a well-known edible alga that has been traditionally consumed in Asian countries, including China, Korea and Japan [1,2,3,4]. *Enteromorpha clathrata* polysaccharide (ECP) has been proposed as a novel drug candidate to treat obesity, constipation and hyperlipidemia [3,5,6]. However, what effect it has on inflammatory bowel diseases has not been investigated. Our study demonstrates for the first time a beneficial effect of ECP on ulcerative colitis and provides a possible basis for understanding its therapeutic mechanisms from the perspective of symbiotic gut bacteria *P. distasonis*. Our study paves the way for the development of ECP as a new marine drug candidate for the treatment of inflammatory bowel diseases such as ulcerative colitis and Crohn’s disease.

Our study has two limitations. First, due to the experimental design, we were not able to determine whether the beneficial effects of ECP on gut microbiota was a cause or consequence of the attenuated colitis. Therefore, future studies using fecal microbiota transplantation and germ-free mice are encouraged to further explore this question. Second, our study could only provide a possible basis for understanding the therapeutic mechanisms of ECP from the perspective of symbiotic gut bacteria *P. distasonis*. This is partially because we failed to isolate any *P. distasonis* strains from the ECP-treated mice. It is hypothesized that ECP could be fermented in vivo by specific *P. distasonis* bacterium. However, more detailed studies are needed to explore this issue.

In addition to *P. distasonis*, *P. goldsteinii* is also recognized as a potential next-generation probiotic candidate due to its protective effect on inflammation [16]. Recently, *P. goldsteinii* was found to attenuate ulcerative colitis in mice treated with DSS [17]. Similarly, *Alistipes finegoldii* was also demonstrated to have a protective effect on DSS-induced colitis [18]. Our study indicated that ECP could increase the abundances of *Alistipes* spp. and *Parabacteroides* spp. in DSS-fed mice during alleviation of colitis symptoms. Although we focused on *P. distasonis* in our present study, we do not rule out the possibility that the anti-colitis effect of ECP may be mediated by other probiotic bacteria in the gut, such as *P. goldsteinii* and *A. finegoldii*.

Intestinal bile acids play a pivotal role in the pathogenesis of inflammatory bowel diseases [19]. *P. distasonis* has been suggested to possess a wide range of bile acid conversion functions [20]. Previous studies have indicated that secondary bile acids, including ursodeoxycholic acid and lithocholic acid, could protect against intestinal inflammation by inhibiting the apoptosis of epithelial cells and restoring the integrity of the mucosal layer [21]. Further investigations are therefore needed to explore the effect of ECP on the metabolism of bile acids during alleviation of ulcerative colitis.

## 4. Materials and Methods

### 4.1. Chemicals and Reagents

The *Enteromorpha clathrata* polysaccharide (ECP) was used and prepared as previously described [1,5]. Tryptone, peptone, yeast extract and Tween 80 were obtained from Sigma (Shanghai, China). Hemin and L-cysteine hydrochloride were purchased from Sangon Biotech (Shanghai, China). All other chemicals of analytical grade were acquired from Sinopharm Chemical (Shanghai, China).

### 4.2. Animals and Treatment

The animal experiments in the present study were approved and supported by the Ethical Committee of Ocean University of China, School of Medicine and Pharmacy (Permission No. OUC-2021-0301-02) and complied with the Guide for the Care and Use of Laboratory Animals (National Academies Press, 8th edition, 2011). All the animals were obtained from Beijing Vital River Laboratory Animal Technology Co. Ltd. (Beijing, China) (Certificate No. SCXK (Jing) 2016-0011). 

In animal experiment 1, a total of 24 eight-week-old male C57BL/6J specific pathogen-free (SPF) mice were used to test the anti-colitis effect of ECP. The mice were divided into three groups: the normal control group (NC, *n* = 8), the model group (MD, *n* = 8) and the ECP treatment group (ECP, *n* = 8). ECP was dissolved in phosphate-buffered saline (PBS) and was given at a dosage of 100 mg/kg/day by gavage. Mice in MD group and ECP group were given 2.0% (*w*/*v*) DSS (MP Biomedicals LLC, Solon, USA) in the daily drinking water for 8 consecutive days. All mice were humanely sacrificed on the eleventh day and the colon and cecum were collected for further experiment. 

In animal experiment 2, a total of 24 eight-week-old male C57BL/6J specific pathogen-free (SPF) mice were used to test the anti-colitis effect of *P. distasonis* F1-28. The mice were divided into three groups: the normal control group (NC, *n* = 8), the model group (MD, *n* = 8) and the *P. distasonis* F1-28 treatment group (PD, *n* = 8). *P. distasonis* F1-28 was dissolved in phosphate-buffered saline (PBS) and was given at a dosage of about 2.0 × 10^7^ colony-forming units (CFUs)/day/mouse by gavage. Mice in MD group and ECP group were given 2.0% (*w*/*v*) DSS in the daily drinking water for 7 consecutive days. All mice were humanely sacrificed on the ninth day and the colon and cecum were collected for further experiment.

The body weight and the stool morphology of the mice were monitored every day. H&E staining and Alcian blue staining of the colon tissues were performed as previously described [13,22,23]. The symptom score and histopathological colon score analyses were performed as described elsewhere [13,24,25].

### 4.3. In Vitro Fermentation of P. distasonis F1-28

The VI medium was applied to investigate the utilization and fermentation of ECP by *P. distasonis* F1-28 as previously described [16,17]. In this in vitro fermentation experiment, *P. distasonis* F1-28 was grown in the basic VI medium that contained no extra carbon source (NC group) and the basic medium that contained ECP as the primary carbon source (ECP group). The CFUs of *P. distasonis* F1-28 was checked at different time points. The SCFAs levels in the two media were analyzed using the methods described elsewhere [26,27].

### 4.4. High-Throughput Sequencing and Bioinformatic Analyses

The metagenomic DNA of the gut microbiota were extracted using the commercial Qiagen QIAamp DNA Stool Mini Kit (Hilden, Germany) from the cecum samples. The 16S V3-V4 hypervariable gene regions were specifically amplified using the well-established universal primers 338F (ACTCCTACGGGAGGAGCAG) and 806R(GGACTACHVGGGTWTCTAAT). The amplicons were quality-checked and sequenced on an Illumina PE300 platform from Majorbio Bio-pharm Biotechnology Co., Ltd. (Shanghai, China). Bioinformatic analyses of the sequencing data (Venn diagram, PCA score plot, NMDS score plot and heatmap analyses) were conducted using the online Majorbio Cloud Platform (www.majorbio.com (accessed on 27 July 2022)). The LEfSe analysis was performed as previously described [26,27]. Only bacterial taxa with an LDA score of above 4.0 and 3.0 were listed. 

### 4.5. Statistical Analyses

Data were expressed as mean ± SEM. Statistical analyses were performed using Student t-test and ANOVA with post-hoc Tukey’s tests (GraphPad Prism for Windows 8.0; GraphPad Software Inc, San Diego, CA, USA). The results were considered statistically significant at *p* < 0.05. * *p* < 0.05 versus NC group; ** *p* < 0.01 versus NC group; ^#^ *p* < 0.05 versus MD group; ^##^ *p* < 0.01 versus MD group.

## 5. Conclusions

In conclusion, dietary ECP improved ulcerative colitis and ameliorated mucosal damage in DSS-fed mice. ECP changed the structure of the gut microbiota in diseased mice by increasing the abundance of probiotic bacteria *Parabacteroides* spp. In vitro, ECP promoted the growth of anti-colitis bacterium *P. distasonis* F1-28 and increased its production of SCFAs. Our study demonstrates a therapeutic effect of ECP on DSS-induced ulcerative colitis and provides a possible basis for understanding its mechanisms from the perspective of symbiotic gut bacteria *P. distasonis*.

## Data Availability

The data presented in this study are available on request from the corresponding authors.

## References

[1] Shang Q., Wang Y., Pan L., Niu Q., Li C., Jiang H., Cai C., Hao J., Li G., Yu G. (2018). Dietary polysaccharide from *Enteromorpha clathrata* modulates gut microbiota and promotes the growth of *Akkermansia muciniphila*, *Bifidobacterium* spp. and *Lactobacillus* spp.. Mar. Drugs.

[2] Cheong K.L., Yu B., Chen J., Zhong S. (2022). A comprehensive review of the cardioprotective effect of marine algae polysaccharide on the gut microbiota. Foods.

[3] Tang Z., Gao H., Wang S., Wen S., Qin S. (2013). Hypolipidemic and antioxidant properties of a polysaccharide fraction from *Enteromorpha prolifera*. Int. J. Biol. Macromol..

[4] Shang Q., Jiang H., Cai C., Hao J., Li G., Yu G. (2018). Gut microbiota fermentation of marine polysaccharides and its effects on intestinal ecology: An overview. Carbohydr. Polym..

[5] Wei J., Zhao Y., Zhou C., Zhao Q., Zhong H., Zhu X., Fu T., Pan L., Shang Q., Yu G. (2021). Dietary polysaccharide from *Enteromorpha clathrata* attenuates obesity and increases the intestinal abundance of butyrate-producing bacterium, *Eubacterium xylanophilum*, in mice fed a high-fat diet. Polymers.

[6] Ren X., Liu L., Gamallat Y., Zhang B., Xin Y. (2017). *Enteromorpha* and polysaccharides from Enteromorpha ameliorate loperamide-induced constipation in mice. Biomed. Pharmacother..

[7] GBD 2017 Inflammatory Bowel Disease Collaborators (2020). The global, regional, and national burden of inflammatory bowel disease in 195 countries and territories, 1990-2017: A systematic analysis for the Global Burden of Disease Study 2017. Lancet Gastroenterol. Hepatol..

[8] Kudelka M.R., Stowell S.R., Cummings R.D., Neish A.S. (2020). Intestinal epithelial glycosylation in homeostasis and gut microbiota interactions in IBD. Nat. Rev. Gastroenterol. Hepatol..

[9] Lavelle A., Sokol H. (2020). Gut microbiota-derived metabolites as key actors in inflammatory bowel disease. Nat. Rev. Gastroenterol. Hepatol..

[10] Schirmer M., Garner A., Vlamakis H., Xavier R.J. (2019). Microbial genes and pathways in inflammatory bowel disease. Nat. Rev. Microbiol..

[11] Chassaing B., Aitken J.D., Malleshappa M., Vijay-Kumar M. (2014). Dextran sulfate sodium (DSS)-induced colitis in mice. Curr. Protoc. Immunol..

[12] Wirtz S., Popp V., Kindermann M., Gerlach K., Weigmann B., Fichtner-Feigl S., Neurath M.F. (2017). Chemically induced mouse models of acute and chronic intestinal inflammation. Nat. Protoc..

[13] Pan L., Fu T., Cheng H., Mi J., Shang Q., Yu G. (2022). Polysaccharide from edible alga *Gloiopeltis furcata* attenuates intestinal mucosal damage by therapeutically remodeling the interactions between gut microbiota and mucin *O*-glycans. Carbohydr. Polym..

[14] Ezeji J.C., Sarikonda D.K., Hopperton A., Erkkila H.L., Cohen D.E., Martinez S.P., Cominelli F., Kuwahara T., Dichosa A.E.K., Good C.E. (2021). *Parabacteroides distasonis*: Intriguing aerotolerant gut anaerobe with emerging antimicrobial resistance and pathogenic and probiotic roles in human health. Gut Microbes.

[15] Cuffaro B., Assohoun A.L.W., Boutillier D., Súkeníková L., Desramaut J., Boudebbouze S., Salomé-Desnoulez S., Hrdý J., Waligora-Dupriet A.-J., Maguin E. (2020). In vitro characterization of gut microbiota-derived commensal strains: Selection of *Parabacteroides distasonis* strains alleviating TNBS-induced colitis in mice. Cells.

[16] Cui Y., Zhang L., Wang X., Yi Y., Shan Y., Liu B., Zhou Y., Lü X. (2022). Roles of intestinal Parabacteroides in human health and diseases. FEMS Microbiol. Lett..

[17] Gerkins C., Oliero M., Hajjar R., Rendos H.V., Fragoso G., Calvé A., Diop K., Routy B., Santos M. (2022). The modulation of intestinal inflammation by parabacteroides goldsteinii in dextran sodium sulfate induced colitis in mice. Gut.

[18] Dziarski R., Park S.Y., Kashyap D.R., Dowd S.E., Gupta D. (2016). Pglyrp-regulated gut microflora *Prevotella falsenii*, *Parabacteroides distasonis* and *Bacteroides eggerthii* enhance and *Alistipes finegoldii* attenuates colitis in mice. PLoS ONE.

[19] Tiratterra E., Franco P., Porru E., Katsanos K.H., Christodoulou D.K., Roda G. (2018). Role of bile acids in inflammatory bowel disease. Ann. Gastroenterol..

[20] Wang K., Liao M., Zhou N., Bao L., Ma K., Zheng Z., Wang Y., Liu C., Wang W., Wang J. (2019). Parabacteroides distasonis Alleviates Obesity and Metabolic Dysfunctions via Production of Succinate and Secondary Bile Acids. Cell Rep..

[21] Lajczak-McGinley N.K., Porru E., Fallon C.M., Smyth J., Curley C., McCarron P.A., Tambuwala M.M., Roda A., Keely S.J. (2020). The secondary bile acids, ursodeoxycholic acid and lithocholic acid, protect against intestinal inflammation by inhibition of epithelial apoptosis. Physiol. Rep..

[22] Yang D., Jacobson A., Meerschaert K.A., Sifakis J.J., Wu M., Chen X., Yang T., Zhou Y., Anekal P.V., Rucker R.A. (2022). Nociceptor neurons direct goblet cells via a CGRP-RAMP1 axis to drive mucus production and gut barrier protection. Cell.

[23] Tawfiq R.A., Nassar N.N., Hammam O.A., Allam R.M., Elmazar M.M., Abdallah D.M., Attia Y.M. (2022). Obeticholic acid orchestrates the crosstalk between ileal autophagy and tight junctions in non-alcoholic steatohepatitis: Role of TLR4/TGF-β1 axis. Chem. Biol. Interact..

[24] Zhou J., Li M., Chen Q., Li X., Chen L., Dong Z., Zhu W., Yang Y., Liu Z., Chen Q. (2022). Programmable probiotics modulate inflammation and gut microbiota for inflammatory bowel disease treatment after effective oral delivery. Nat. Commun..

[25] Yu J., Zhang D., Liang Y., Zhang Z., Guo J., Chen Y., Yan Y., Liu H., Lei L., Wang Z. (2020). Licorice-Yuanhua Herbal Pair Induces Ileum Injuries Through Weakening Epithelial and Mucous Barrier Functions: Saponins, Flavonoids, and Di-Terpenes All Involved. Front. Pharmacol..

[26] Fu T., Pan L., Shang Q., Yu G. (2021). Fermentation of alginate and its derivatives by different enterotypes of human gut microbiota: Towards personalized nutrition using enterotype-specific dietary fibers. Int. J. Biol. Macromol..

[27] Fu T., Zhou L., Fu Z., Zhang B., Li Q., Pan L., Zhou C., Zhao Q., Shang Q., Yu G. (2022). Enterotype-specific effect of human gut microbiota on the fermentation of marine algae oligosaccharides: A preliminary proof-of-concept in vitro study. Polymers.

